# Association between periodontal disease and schizophrenia: a bidirectional two-sample Mendelian randomization study

**DOI:** 10.1038/s41598-024-65181-3

**Published:** 2024-07-29

**Authors:** Hongliang Cao, Hao Wu, Pengyu Wang, Haiyang Zhang, Song Wang

**Affiliations:** 1 Department of Urology II, The First Hospital of Jilin University, Changchun, 130021 China; 2 Department of Prosthodontics, Hospital of Stomatology, Jilin University, Changchun, 130021 China

**Keywords:** Periodontal disease, Schizophrenia, Mendelian randomization, Bi-directional causal effects, Psychology, Diseases, Medical research, Risk factors

## Abstract

The connection between periodontal disease (PD) and schizophrenia (SCZ) has been reported in observational studies, but it remains unclear. This research aims to examine the bidirectional causal impacts between PD and SCZ. The FinnGen consortium supplied summarized data on PD for 346,731 individuals (87,497 cases and 259,234 controls) of Finnish ancestry, and information on SCZ was acquired from the OpenGWAS repository, encompassing 127,906 individuals (52,017 cases and 75,889 controls) of European ancestry. Next, we conducted Mendelian randomization (MR) analysis to establish a causal inference relationship between PD and SCZ. The inverse variance weighted (IVW) method was utilized as the primary analysis. Additionally, some sensitivity analyses were utilized to verify the stability of the results. The analysis of IVW results indicated no impact of PD on SCZ (IVW OR = 1.10, 95% CI 0.97–1.24, P = 0.14). Nevertheless, the inverse relationship between PD and SCZ was identified through reverse MR analysis (IVW OR = 1.03, 95% CI 1.01–1.05, P = 0.002). The findings from MR-Egger, weighted median, simple mode, and weighted mode approaches aligned with the outcomes of the IVW method. Based on sensitivity analyses, horizontal pleiotropy is unlikely to distort causal estimates. This study presented the initial proof of a genetic causal relationship between SCZ and PD, albeit with a minimal impact. Further exploration is needed to gain a deeper understanding of this relationship. Furthermore, no genetic causal relationship between PD and SCZ was identified.

## Introduction

Periodontal disease, also known as PD, is a multifaceted inflammatory condition involving multiple microorganisms that result in the erosion of the alveolar bone and eventual tooth decay. Periodontal disease mainly includes gingivitis and periodontitis, which cause significant medical and economic burdens to society^1–3^. According to previous studies, PD has been associated with the emergence of various health conditions such as heart disease, kidney disease, diabetes, lung disease, colon cancer, pancreatic cancer, premature pregnancy, impotence, Alzheimer's disease, and rheumatoid arthritis^4^. Schizophrenia, also known as SCZ, is a long-lasting mental illness characterized by a diverse genetic and neurobiological foundation that affects the early growth of the brain. It manifests as a mixture of psychotic indications, including hallucinations, delusions, disorganization, and impairments in motivation and cognition^5^.

Numerous studies have identified a connection between PD and SCZ. A retrospective cohort study found that out of 3610 individuals newly diagnosed with SCZ, 2373 (65.7%) experienced treated PD within the 1-year follow-up period^6^. In a case–control study with 100 participants, consisting of 50 individuals diagnosed with SCZ and 50 control subjects, there was a notable rise in the likelihood of PD within the case group (54% in the case group compared to 12% in the control group, P < 0.001)^7^. In line with these findings, numerous other studies have similarly discovered that individuals with SCZ have a highly elevated likelihood of developing PD^8–10^. Nevertheless, insufficient evidence exists to establish a substantial correlation between PD and SCZ^11^. PD and SCZ are potentially influenced by various factors, including age, sex, race, socioeconomic status, smoking, alcohol consumption, physical activity, cardiovascular, education, and diabetes^12–19^. There have been no investigations into the genetic causality connecting PD and SCZ.

Mendelian randomization (MR) is an investigative technique that explores the cause-and-effect connections between risk factors and disease outcomes by utilizing single nucleotide polymorphisms (SNPs) as instrumental variables (IVs)^20,21^. MR can address potential biases like confounders or reverse causation and investigate the causality between risk factors and the disease^22,23^. This study utilized a two-sample MR approach by employing summary statistics from extensive genome-wide association studies (GWASs) on PD and SCZ. We aimed to investigate the reciprocal causal impact of PD and SCZ.

## Approaches and procedures

### Study design

**Figure 1:**
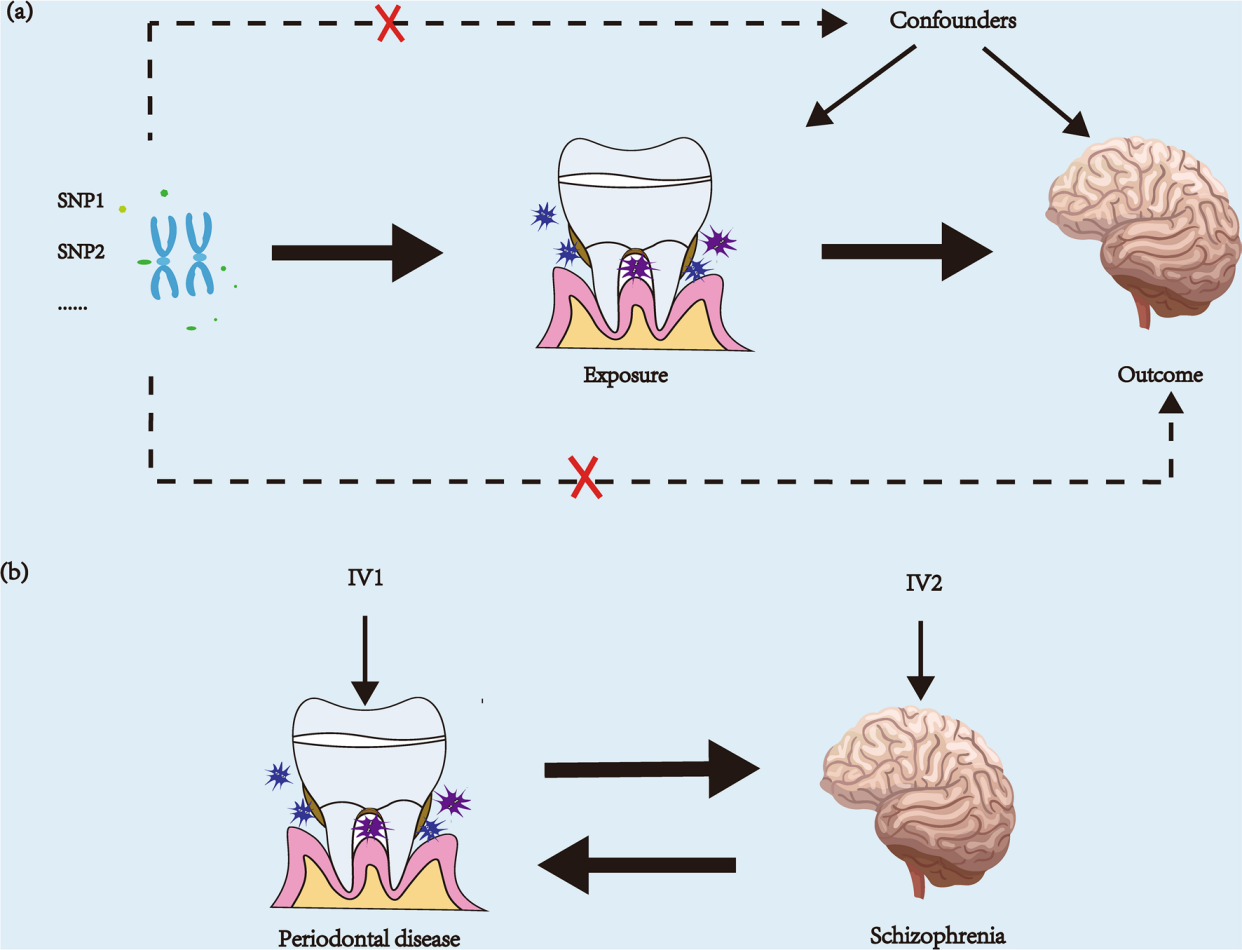
(**a**) Three key assumptions of MR. (**b**) The casual effects of PD on SCZ and SCZ on PD are explored. MR: Mendelian randomization; PD: periodontal disease; SCZ: schizophrenia.

A bidirectional two-sample Mendelian randomization (MR) study was employed to establish the causal relationship between PD and SCZ. The studies on MR rely on instrumental variables (IVs) and employ Single Nucleotide Polymorphisms (SNPs) to investigate the causal connection between exposure and outcome. The foundation of MR is based on three main principles: (1) the correlation hypothesis, which states a strong correlation with exposure; (2) the exclusivity hypothesis, which is unrelated to the outcome; (3) the independence hypothesis, which is unrelated to confounding factors^24,25^. Figure 1 shows the layout of this MR.

### GWAS statistics source

The GWAS associated with PD was provided by the Release 9 of FinnGen research project of European descent (available from: https://www.finngen.fi/en). After adjusting for age, gender, genetic relatedness, genotyping batch, and the first 10 principal components, the analysis was comprised with 346,731 individuals (87,497 cases and 259,234 controls). Periodontal disease cases were diagnosed based on the K05 criteria in the International Classification of Diseases 10 (ICD-10) Revision codes and codes 523 in ICD-8 and ICD-9. Additionally, diseases of oral cavity, salivary glands and jaws were not included in the control group. FinnGen is a comprehensive collaboration between the public and private sectors to gather and examine genetic and medical information from 500,000 individuals in Finnish biobanks^26^. The OpenGWAS repository (available from: https://gwas.mrcieu.ac.uk) is a collection of openly accessible datasets that offer summarized statistical information for SCZ, encompassing 127,906 individuals (52,017 cases and 75,889 controls) of European ancestry^27^.

### Selection of genetic instrumental variables

To investigate the reciprocal causal relationship between PD and SCZ, we employed two sets of instrumental variables (IVs) for each condition. As potential instrumental variables (IVs), we chose single nucleotide polymorphisms (SNPs) that were highly correlated with PD and had significant genome-wide importance (P-value < 5 × 10^−8^). Following this, we eliminate linkage disequilibrium (LD) and establish the threshold as r^2^ < 0.001, kb = 10,000. Nevertheless, the number of autonomous SNPs at the P-value < 5 × 10^−8^ threshold is insufficient to carry out MR for PD. As a result, we relaxed the threshold as following criteria. We selected SNPs that exhibited genome-wide significance (P-value < 5 × 10^−6^ for PD, P-value < 5 × 10^−8^ for SCZ), linkage disequilibrium (LD), and an r^2^ < 0.001 threshold within a 10,000 kb window, which has been used by other researches^28^. We removed palindromic variants for incompatible alleles. The strength of the selected SNPs was evaluated by calculating the F-statistic with following equation:

$$F=\frac{{R}^{2}(N-2)}{1-{R}^{2}}$$

where R^2^ is the portion of exposure variance explained by the IVs, N is the sample size. SNPs were considered for inclusion if the F-statistic was great than or equal to 10^29^. In addition, we utilized PhenoScanner (website: www.phenoscanner.medschl.cam.ac.uk) to examine if these SNPs had any connections with the potential influencing factors such as smoking, alcohol consumption, level of physical activity, body mass index (BMI), and diabetes^30^. SNPs related to any of these potential confounders were removed. Before conducting each MR analysis, the MR-PRESSO test was utilized to eliminate any outliers present in the data^31^. We performed the Steiger test to prevent reverse causality and incorporated SNPs with TRUE results^32^. For the subsequent MR study, the final IVs comprised the carefully selected SNPs.

### Statistical analysis

**Figure 2:**
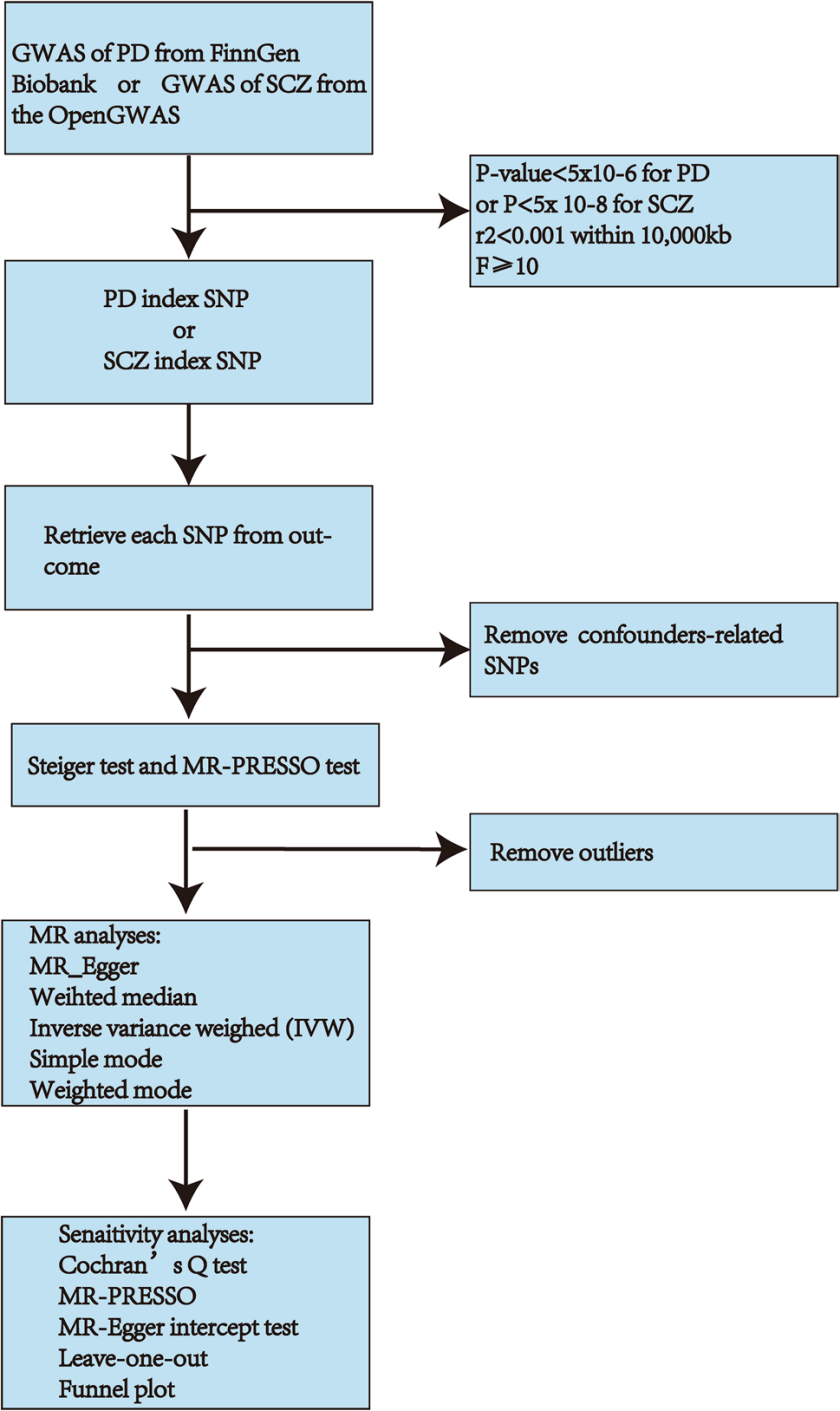
Workflow chart of MR revealing association between PD and SCZ. PD: periodontal disease; SCZ: schizophrenia; SNP: Single Nucleotide Polymorphisms; MR: Mendelian randomization.

The study utilized five distinct MR techniques: MR Egger, weighted median, random-effect inverse-variance weighted (IVW), simple mode, and weighted mode. The IVW estimates were enhanced using four additional methods to improve their robustness in a broader range of scenarios despite being less efficient and resulting in more comprehensive confidence intervals (CIs)^33^. The resulting estimate was represented by the slope of the weighted regression in the IVW analysis, which was obtained by constraining the intercept to zero and analyzing the SNP-outcome effects concerning the SNP exposure effects^34^. To assess the heterogeneity of MR results, Cochran’s Q test was employed, utilizing IVW and MR Egger methods^35^. Moreover, the Pleiotropy Residual Sum and Outlier methods (MR-PRESSO) were employed to evaluate and rectify horizontal pleiotropy. In addition, a Leave-one-out analysis was conducted to assess whether a single SNP impacted or biased the MR estimate^36^. A funnel plot was implemented to assess the likely directional pleiotropy. A significance level of less than 0.05 was deemed statistically significant. Figure 2 displays the demonstrated workflow of MR. Statistical analyses were performed using the TwoSampleMR R package (version 0.5.7) and MR-PRESSO (version 1.0) in R software 4.3.1 (website: https://cloud.r-project.org).

### Ethics statement

The GWAS data used in this MR were all publicly available, and ethical permission and informed consent had been provided in the original study.

## Results

### The impact of PD on SCZ

We used 20 separate SNPs with P-values below 5 × 10^−6^ and an r^2^ < 0.001 thresholds within a 10,000 kb window as instrumental variables for PD. Nevertheless, one single nucleotide polymorphism (SNP) associated with PD was not present in the summary statistic of SCZ and was consequently omitted. Three SNPs of palindromic variants were removed for incompatible alleles. All of the F-statistic values exceeded 10. The MR-PRESSO result showed no outliers. Two single nucleotide polymorphisms (SNPs) were linked to confounding factors and eliminated. Supplementary Table S1 provides comprehensive details regarding the last 14 SNPs for PD.

**Figure 3:**
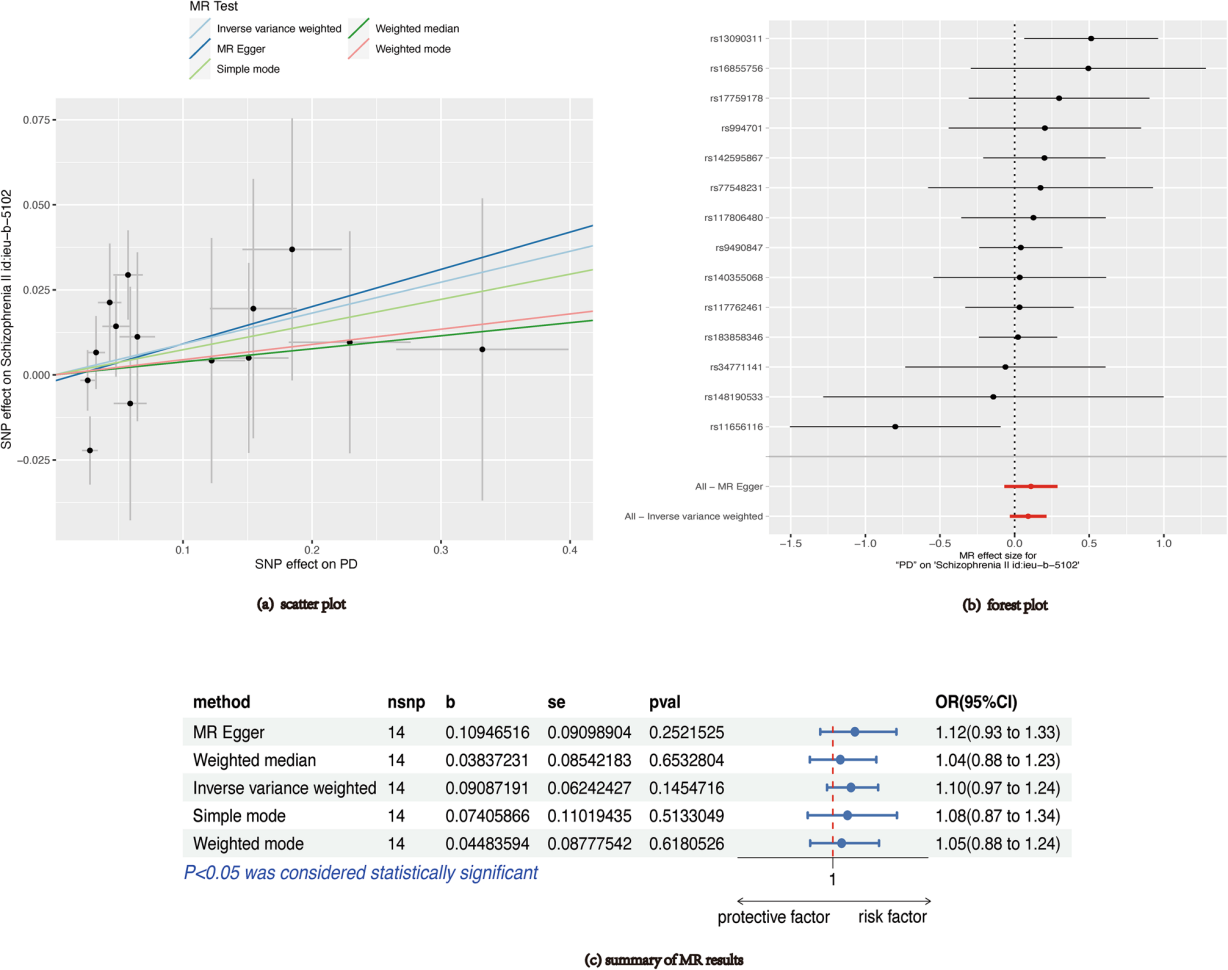
MR results of PD on SCZ. PD: periodontal disease; SCZ: schizophrenia; MR: Mendelian randomization.

Figure 3 displays the MR estimates of various techniques. In general, there were no direct connections between PD and SCZ vulnerability. According to the main findings of IVW, there was no significant association between elevated risk of PD and an increased risk of SCZ (OR = 1.10, 95% CI 0.97–1.24, P = 0.14). Furthermore, the MR-Egger, the weighted median, the weighted mode, and the simple mode approaches exhibited consistent findings. Figure 3 displays the scatter plot illustrating the effect sizes of SNPs for PD and SCZ. Based on Cochran's Q test (Supplementary Table 2), there was no variation among the individual SNP. Based on the findings from the MR-Egger intercept and MR-PRESSO global test (Supplementary Table S2), it is improbable that horizontal pleiotropy would distort the causal relationship between PD and SCZ. The causal estimates of PD were not driven by any individual SNP, as indicated by the leave-one-out analysis (Supplementary Fig. S1).

### The impact of SCZ on PD

For SCZ, we selected 158 autonomous SNPs with P-values lower than 5 × 10^−8^ and an r^2^ < 0.001 threshold within a 10,000 kb range as instrumental variables (IVs). Nevertheless, four SNPs associated with SCZ were not present in the summary statistic of PD and were therefore omitted. Incompatible alleles led to the removal of twenty-five palindromic variant SNPs. The MR-PRESSO analysis indicated an outlier (rs113264400) which was then removed. A total of twenty-seven SNPs were linked to confounding factors and subsequently eliminated. All of the F-statistic values exceeded 10. Supplementary Table S3 provided comprehensive details regarding the final 101 SNPs for SCZ.

**Figure 4:**
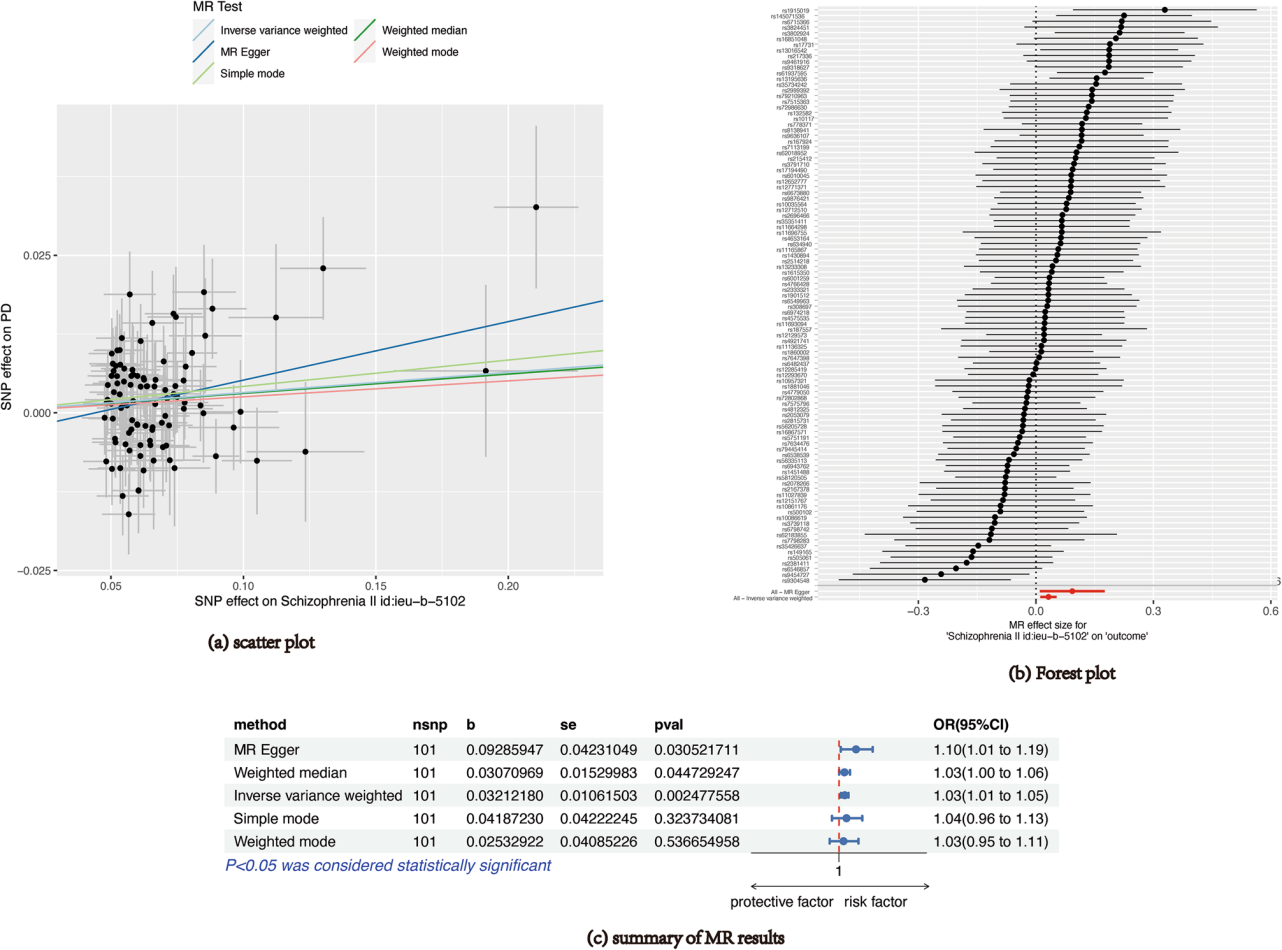
MR results of SCZ on PD. SCZ: schizophrenia; PD: periodontal disease; MR: Mendelian randomization.

Figure 4 displays the MR estimates of various techniques. In general, there were connections between SCZ and the risk of PD. The main findings from IVW indicated a higher likelihood of developing SCZ was statistically linked to a greater chance of developing PD (OR = 1.03, 95% confidence interval 1.01–1.05, P-value = 0.002). Furthermore, the MR-Egger, the Weighted Median, the fundamental mode, and the Weighted Mode approaches exhibited consistent findings. Figure 4 displays the scatter plot illustrating the effect sizes of SNPs for SCZ and PD. Based on Cochran's Q test (Supplementary Table S4), there was no heterogeneity among the individual SNP. Based on the findings from the MR-Egger regression and MR-PRESSO global test (Supplementary Tables S4), it is improbable that horizontal pleiotropy would distort the causality between SCZ and PD. The causal estimates of PD were not driven by any individual SNP, as indicated by the leave-one-out analysis (Supplementary Fig. S2).

## Discussion

This study used Mendelian randomization to examine the reciprocal causal connections between PD and SCZ. The findings suggest a favorable correlation between SCZ and the likelihood of PD (IVW OR = 1.03, 95% CI 1.01–1.05, P = 0.002). Furthermore, our findings did not indicate a causal relationship between PD and SCZ (IVW OR = 1.10, 95% CI 0.97–1.24, P = 0.14). The results offer a valuable understanding of the involvement of SCZ in the development of PD, which could impact the creation of preventive and treatment approaches for PD in individuals with SCZ.

Previous research has mainly found a connection between SCZ and the existence of PD through observational studies. In previous studies conducted by Kai-Fang Hu, a significant and separate correlation was found between higher PD rates and individuals diagnosed with SCZ^6^. In the UK Biobank study (2007–2010), an examination of initial data showed that individuals with psychosis had a more significant percentage of PD in comparison to the overall population (21.3% vs. 14.8%, prevalence ratio 1.40, 95% CI 1.26–1.56)^37^. Nevertheless, potential explanations for the observed associations include residual confounding, reverse causation, or a combination of both. The current research indicates that SCZ has a causal impact on PD, albeit with a relatively minor magnitude of the effect estimation. Significantly, this is the initial MR to investigate the association between SCZ and an elevated likelihood of PD. Nonetheless, the underlying mechanisms of SCZ and PD remain unknown^11,38^. Research has demonstrated that individuals experiencing a psychotic episode in SCZ exhibit elevated levels of inflammatory cytokines in their bloodstream, such as interleukin-12, interferon-gamma, tumor necrosis factor-alpha, and C-reactive protein^39^. Hence, the mild, persistent inflammatory condition of SCZ might play a role in immune system irregularities, making individuals with SCZ more susceptible to systemic illnesses, including PD^40^.

Furthermore, the causal impact of PD on SCZ remained elusive to us. Although there is limited research on the influence of PD on SCZ, several articles have documented the correlation between PD and various mental illnesses, such as major depression, bipolar disorder, Alzheimer's disease, and substance use disorders. A potential connection could be clarified by chronic systemic inflammation leading to neuroinflammation^11^. Four suggested direct causal mechanisms include the escape of microbes and metabolites, neuroinflammation, the central nervous system's signaling, and the neurohormones' response. Inflammation through a host response is a recurring theme among these mechanisms^38^. Additional investigation is required to examine the impact of PD on psychiatric disorders such as SCZ.

There are multiple advantages to the current research. Initially, it was the primary study to investigate the impact of SCZ on PD by utilizing extensive GWAS data from Finngen Biobank and the OpenGWAS. Furthermore, we employed various reliable techniques to acquire the MR effects, including MR-PRESSO and the Steiger test. Moreover, we evaluated the presence of horizontal pleiotropy. Ultimately, by employing the two-sample MR analysis, we successfully pinpointed SCZ as a contributing factor to the risk of PD^41^.

There are certain constraints in the current investigation. Initially, the increased risk discovered in our study was marginal and, possibly, of limited clinical relevance. Additional research is necessary to support the findings of our present study. Furthermore, the results may have limited relevance to individuals of non-European descent due to the exclusive inclusion of participants with European ancestry. Moreover, the GWAS data may give rise to potential nonlinear relationships or stratification effects^23^. Finally, there is a possibility of overlap of included subjects with the FinnGen study. As Trubetskoy et al. notes, there are Finnish subjects in the SCZ study^27^. However, we were unable to assess this.

## Conclusion

In conclusion, our findings indicate that there is a minimal and probably limited clinical relevance to the genetic influence of SCZ on PD. Further investigation is required to gain a deeper understanding of this relationship. Furthermore, we were unable to ascertain the genetic causal effect of PD on SCZ.

### Supplementary Information

Supplementary Figures.

Supplementary Tables.

## Data Availability

The GWAS associated with PD was provided by FinnGen Biobank (https://www.finngen.fi/en). The OpenGWAS repository (https://gwas.mrcieu.ac.uk) offers summarized statistical information for SCZ from PSYCHIATRIC GENOMICS CONSORTIUM (PGC). The authors thank all individuals who dedicated to these GWAS summary statistics publicly available.
