# Supplementary material for: Association between periodontal disease and schizophrenia: a bidirectional two-sample Mendelian randomization study
Source: Sci Rep. 2024 Jul 29;14:17391. doi: 10.1038/s41598-024-65181-3 (PMC11286959; doi:10.1038/s41598-024-65181-3)
Supplement: Supplementary file 1 — Supplementary Figures. [file 41598_2024_65181_MOESM1_ESM.docx]

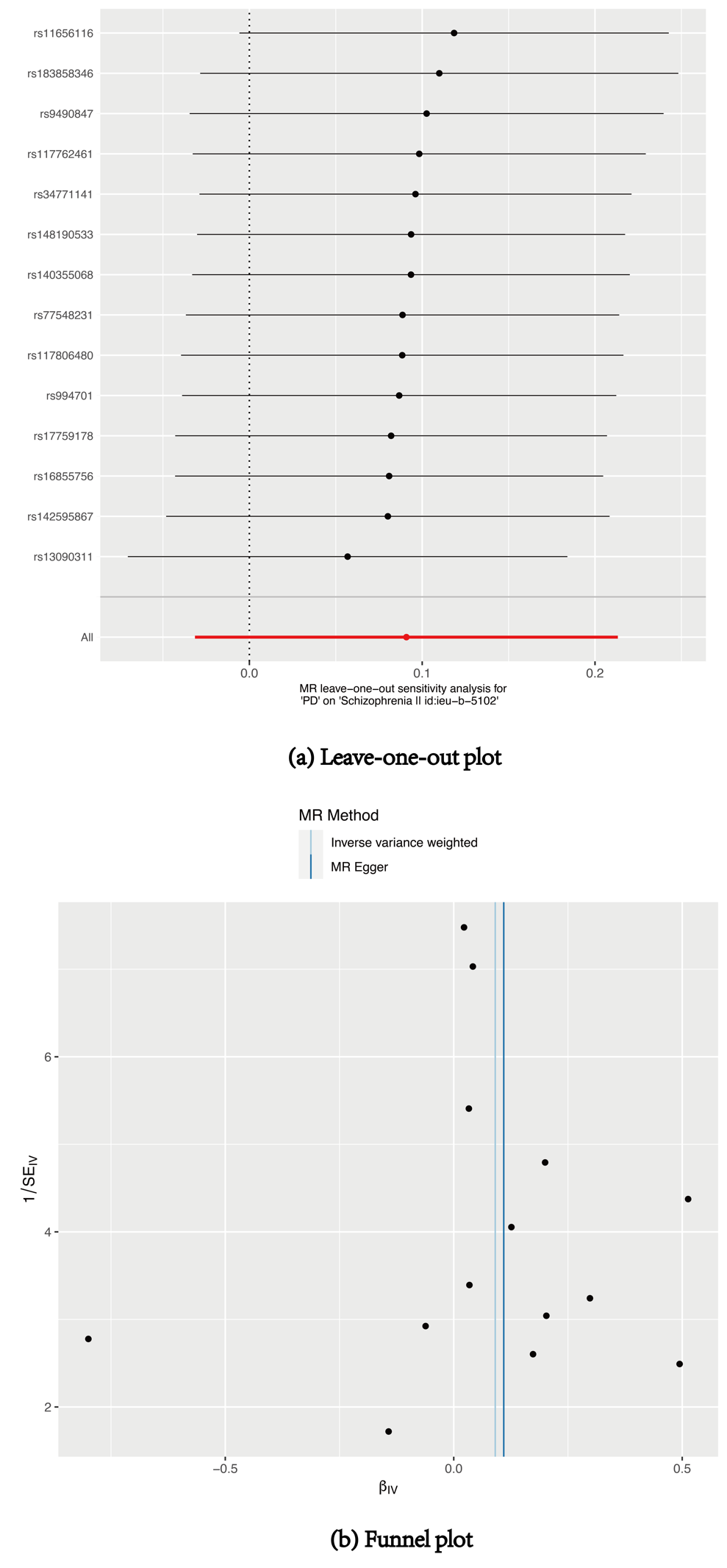


**Supplementary figure 1** (a) Leave-one-out (b) Funnel plot Abbreviation PD: periodontal disease


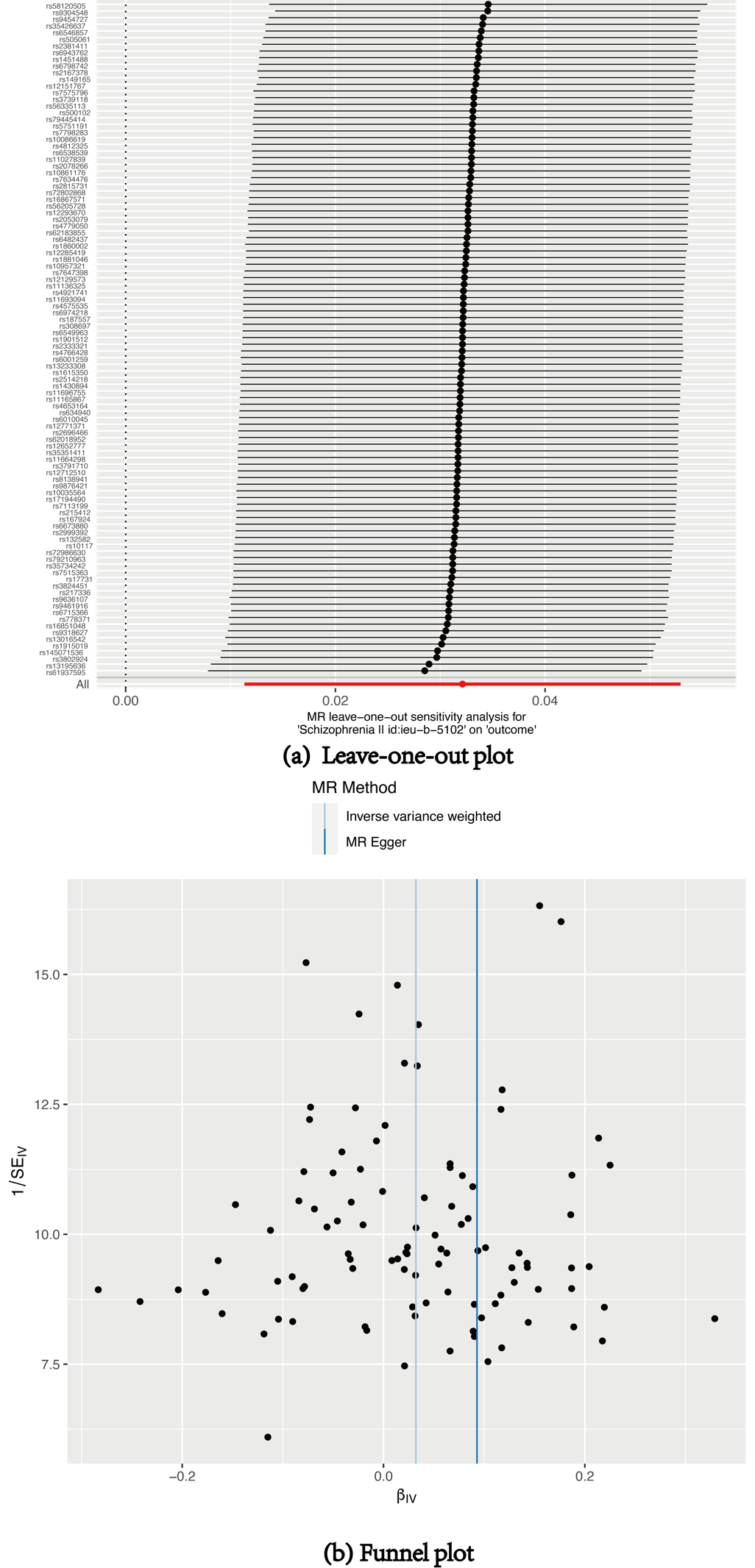


**Supplementary figure 2** (a)Leave-one-out (b) Funnel plot Abbreviation PD: periodontal disease
